# Construction of a new smooth support vector machine model and its application in heart disease diagnosis

**DOI:** 10.1371/journal.pone.0280804

**Published:** 2023-02-09

**Authors:** Jianjian Wang, Feng He, Shouheng Sun

**Affiliations:** 1 School of Information, Beijing Wuzi University, Beijing, 101149, China; 2 School of Economics and Management, University of Science and Technology Beijing, Beijing, 100083, China; Minnan Normal University, CHINA

## Abstract

Support vector machine (SVM) is a new machine learning method developed from statistical learning theory. Since the objective function of the unconstrained SVM model is a non-smooth function, a lot of fast optimization algorithms can’t be used to find the solution. Firstly, to overcome the non-smooth property of this model, a new padé33 approximation smooth function is constructed by rational approximation method, and a new smooth support vector machine model (SSVM) is established based on the smooth function. Then, by analyzing the performance of the smooth function, we find that the smooth precision is significantly higher than existing smooth functions. Moreover, theoretical and rigorous mathematical analyses are given to prove the convergence of the new model. Finally, it is applied to the heart disease diagnosis. The results show that the Padé33-SSVM model has better classification capability than existing SSVMs.

## 1. Introduction

With the development of machine learning, support vector machine (SVM) is a new machine learning method in statistical learning theory, and has achieved remarkable results in face recognition, population prediction, image retrieval, data mining and other fields [1–4]. SVM has good generalization performance, and its classifier shows special advantages in solving the pattern recognition problems of small samples, nonlinearity and high dimension, especially in dealing with classification problems [5–7]. At present, the research of SVM mainly includes the following three aspects: First, the improvement of SVM model. Lopez et al. [8] proposed an extension of the non-parallel SVM method and experimentally demonstrated the advantages of achieving the best average performance compared to other SVM methods. Subsequently, Chen et al. [9] improved projective Twin Support Vector Machine (PTSVM) to a novel non-parallel classifier, and experimental evaluation on both synthetic and real-world datasets demonstrated the feasibility and effectiveness of the proposed approach. In literature [10], a new weighted quantum particle swarm optimization (WQPSO) hybrid model is proposed for sequence data clustering based on Smooth Support Vector Machine (SSVM) for classification. The limitation of the proposed work is an increase of computational complexity due to the usage of weighing optimization strategy. Some studies yet to be conducted to reduce the computational complexity. In order to solve the problem that the existing common classification algorithm has poor fault tolerance, identify single lithology and cannot effectively overcome the imbalance between classes, Su et al. [11] proposed an improved Synthetic Minority Over Sampling Technique (SMOTE)to process data sets and proposed a new fuzzy membership function to improve the fuzzy twin support vector machine. Sun et al. [12] proposed a partial binary tree twin support vector machine multi-classification algorithm based on optimal classification features (OCF-PBT-TWSVM) to achieve effective classification of non-stationary transient random signals with edge distortion of tooth profile images, and to meet the requirements of real-time gear vision measurement and distortion compensation accuracy claim. Fang et al. [13] proposed a new model: similarity feature transformation smooth support vector machine based on fuzzy C-means and the experimental results indicate that the proposed model has better performance compared with the conventional SVM model as well as some variants in terms of classification accuracy and training time. In order to solve the problem of low approximation accuracy of S-shaped smooth function adopted by smooth twin support vector machine (STWSVM), a polynomial smooth twin support vector machine model based on Newton Armijo optimization (PSTWSVM-NA) was proposed in [14].

The second is the application of SVM model. Indrawan et al. [15] focused on developing this prediction at the suicidal behavior of psychiatric patients by using smooth support vector machine method, which is very useful for the prevention of psychiatric hospitals. In order to solve the problem of vibration signal recognition of fiber perimeter, Ma et al. [16] proposed a vibration signal recognition method based on SVD and MPSO-SVM, and predicted the fiber signal, achieving good classification results. Wang et al. [17] proposed a new method combining computer numerical simulation method with machine learning to predict deep mineralization. Wang and Dong [18] combined support vector machine (SVM) and ant colony algorithm (ACA) to study the problem of power human accident, and proposed a statistical analysis model for data fitting and prediction of power human accident.

Third, the algorithm for solving the SVM model. In order to solve the excessive consumption of space and time in standard support vector machines, a new method of training support vector machines using boundary points is proposed in [19]. To improve the accuracy of group activity recognition in video, a group activity recognition algorithm based on tensor features and twin support vector machines is proposed in [20]. In order to reduce the error of communication information security situation prediction, Wang et al. [21] designed a support vector machine communication information security situation prediction model based on ant colony optimization algorithm, and compared it with the traditional support vector machine.

In general, support vector machine (SVM) is a new machine learning method based on statistical theory. It has outstanding performance in small sample, high-dimensional and nonlinear problems, and has achieved great success in many fields. Although the conventional support vector machine has many advantages, there are still many problems. In recent years, researchers have a large number of methods to reduce the impact of outliers and noise points on conventional support vector machines. At the same time, many researches have been carried out on how to filter support vectors effectively when dealing with large-scale datasets, and there are generally two aspects that need to be improved. Firstly, the conditional quadratic convex optimization problem restricts the use of some effective optimization algorithms in the field of unconditional optimization; Secondly, when large sample data sets need to be processed, the training time of the algorithm is relatively long and the efficiency is low.

Therefore, to overcome such problems, Lee and Mangasarian [22] first to introduce the Sigmoid integral function into the SVM to smooth the non-differentiable SVM model, and established a smooth support vector machine model (SSVM) based on the Sigmoid function. Thus, many fast optimization algorithms can be used to solve it, which greatly reduces the computational complexity. SVM has strict convexity and infinitely differentiable mathematical characteristics, and introduced efficient unconstrained optimization problems, which attracted many scholars to study SSVM models from different perspectives [23–29], Some proposed different smoothing functions [23–26], while others extended them to the prediction field [16,17]. Other applications of SVM and SSVM are introduced in [27–29].

In conclusion, because the objective function of unconstrained SVM model is not smooth, many fast optimization algorithms can’t solve it. Many scholars [22–26] proposed to solve such problems by approximating the objective function of support vector machine with smooth function. In the research, we found that the performance of SSVM will change with the change of smooth function, that is, the higher the approximation accuracy of smooth function, the better the convergence of the corresponding SSVM model, and thus the higher the classification accuracy. To further explore the smooth function with higher approximation accuracy, based on previous studies, this paper first constructs padé33 rational smooth function to approximate positive sign function through the calculation method of determinant formula and the definition of an orthogonal polynomial of padé type approximation. Secondly, the approximation accuracy of the smooth function and the convergence performance of the model are analyzed and proved, and compared with the existing smooth functions. Finally, through the numerical experiments on heart disease, it is proved that the smooth vector machine model based on the smooth function in this paper can obtain high classification accuracy when used to deal with classification problems.

This paper is organized as follows. First, Section 2 reviews some preliminary knowledge about smooth support vector machine model. Then, a new padé33 smooth function and two theorems are proposed in Section 3. After that, we present numerical examples to illustrate the potential applications of the new models and compare six methods of the model in Section 4 and Section 5. Finally, the concluding remarks and future research directions are given in Section 6.

## 2. Smooth support vector machine model

For binary classification problems [30], the training set includes *m* samples ${\left\{(x_{i},y_{i})\right\}}_{i=1}^{m}$, where ***x***_***i***_∈***R***^***n***^ is the row vector and *y*_*i*_∈{1,−1}. These *m* samples such as ***x***_***i***_ (*i* = 1,⋯,*m*) are represented by matrix ***A***_*m×n*_. ***x***_***1***_**,*x***_***2***_**,*…*,*x***_***m***_ is divided into two categories *A*^+^ and *A*^−^. If ***A***_*i*_ belongs to class *A*^+^, denoted as 1; otherwise, ***A***_*i*_ belongs to class *A*^−^, denoted as -1, that is, the goal of the support vector machine is to divide ***A***_***1***_,***A***_***2***_,…,***A***_***m***_ into two categories: *A*^+^ and *A*^−^. Therefore, the specific classification can be represented by a diagonal matrix ***D*** of type *m*×*n*, where the diagonal element of ***D*** is 1 or -1. Thus, a strongly convex support vector machine (SVM) model modified by this problem is established as follows:

$$\begin{array}{l} \underset{(\omega ,b,\xi )\in R^{n+1+m}}{\min}\;\frac{C}{2}\xi^{T}\xi +\frac{1}{2}(\omega^{T}\omega +b^{2})\\ \;s.t.\;D(A\omega -eb)+\xi \geq e,\xi \geq 0. \end{array}$$ (1)

Where ***ξ*** is the relaxation variable, ***e*** = (1,…,1)^*T*^ is the column vector with all units of 1, *C*>0 is the misdivision penalty parameter, and ***ω*** is the normal vector of the following boundary surface:

$$\left\{ \begin{array}{l} x^{T}\omega -b=1\\ x^{T}\omega -b=-1 \end{array}\right.$$ (2)

In Formula (2), b is the distance from the boundary surface to the origin.

For model (1), on the one hand, when *A*^+^ and *A*^−^ are strictly linearly separable, there is ***ξ*** = 0; ***x***^***T***^***ω***−*b* = 1 and ***x***^***T***^***ω***−*b* = −1 are the boundaries of *A*^+^ and *A*^−^ respectively. At this time, ***x***^***T***^***ω*** = *b* is the classified hyperplane, that is, the plane is located between the two edge interfaces described in (2), as shown in S1 Fig. On the other hand, when *A*^+^ and *A*^−^ are quasilinear separable, then there is ***ξ***>0, which will make: if ***x***^***T***^ = ***A***_***i***_, ***D***_***ii***_ = 1, then ***x***^***T***^***ω***−*b*+***ξ***_***i***_≥1; Otherwise, ***x***^***T***^ = ***A***_***i***_, ***D***_***ii***_ = −1, and ***x***^***T***^***ω***−*b*+***ξ***_***i***_≤−1.

Obviously, model (1) is a quadratic programming problem with constraints. In (1), if

$$\xi ={\left(e-D(A\omega -eb)\right)}_{+}$$ (3)

Here (.)_+_ is a positive sign function, that is, ${\left(x\right)}_{+}={\left({\left(x_{1}\right)}_{+},{\left(x_{2}\right)}_{+},\ldots ,{\left(x_{m}\right)}_{+}\right)}^{T}$, and ${\left(x_{i}\right)}_{+}=\max\{0,x_{i}\},$ *i* = 1,2,…,*m*, Replace (3) in Formula (1) to obtain an unconstrained optimization model:

$$\underset{\omega ,b}{\min}\frac{C}{2}{\left\|{\left(e-D(A\omega -eb)\right)}_{+}\right\|}_{2}^{2}+\frac{1}{2}(\omega^{T}\omega +b^{2})$$ (4)

The objective function of the unconstrained optimization model (4) has the characteristics of strong convexity. Since the minimized objective function contains a non-smooth positive sign function ***ξ*** = (***e−D***(***Aω***−***e****b*))_+_, it cannot be solved by a smooth unconstrained optimization algorithm. Therefore, scholars have proposed different styles of smooth functions to approximate positive sign functions and established SSVM models with sigmoid integral functions, quadratic and quartic polynomials, cubic and quantic spline functions, and padé22 approximation rational functions as smooth functions [22–26]. Since different smooth functions can construct different SSVM models correspondingly, for convenience, the above smooth models are recorded as sigmoid-SSVM, P2 -SSVM, P4-SSVM, T3-SSVM, T5-SSVM, padé22-SSVM respectively. For the convenience of subsequent experiments, a unified model (5) is used to solve the linearly separable case, and the function *f*(*x*) in (5) can be replaced by different smooth functions.


$$\underset{\omega ,b}{\min}\frac{C}{2}{\left\|f{\left(e-D(A\omega -eb)\right)}_{+}\right\|}_{2}^{2}+\frac{1}{2}(\omega^{T}\omega +b^{2})$$ (5)

## 3. Construction and analysis of a new padé33 smooth function

### 3.1 Constructing padé33 rational smooth function approximating (*x*)_+_

**Lemma 1** ([26]) Assuming *k*>0, the positive sign function *x*_+_ = max{0,*x*} can be expanded into uniformly convergent polynomial series on the interval $\left[-\frac{1}{k},\frac{1}{k}\right]$, that is

$$x_{+}=\frac{|x|+x}{2}=L(x,k)=\frac{1}{2k}[\frac{1+k^{2}x^{2}}{2}-\sum_{n=2}^{\infty }\frac{(2n-3)!!}{(2n)!!}{\left(1-k^{2}x^{2}\right)}^{n}]+\frac{x}{2}$$ (6)

Based on (6), the related concepts of the Padé approximation of *f*(*x*) are proposed as follows:

**Definition 1** ([31]): Suppose *f*(*x*) is a power series with *c*_*i*_∈*C* as the coefficient, as follows: $f(x)=c_{0}+c_{1}x+c_{2}x^{2}+\ldots +c_{n}x^{n}+\ldots$. Let $\tilde{q_{n}(x)}$ and $\tilde{p_{m}}(x)$ be polynomials of degree *n* and *m* respectively, if they satisfy: $\tilde{q_{n}(x)}f(x)-\tilde{p_{m}}(x)=Ο(x^{m+n+1})$, then the rational expression $\tilde{p_{m}}(x)/\tilde{q_{n}(x)}$ is called the Padé approximation of the function *f*(*x*), denoted by [m/n]_f(x)_.

According to definition 1, a padé rational approximation of *f*(*x*) can be obtained by approximating the positive sign function derivation, as follows:

$${\left[\mathrm{m/n}\right]}_{f(x)}=\frac{\tilde{p_{m}}(x)}{\tilde{q_{n}}(x)}=\frac{\sum_{i=0}^{n}a_{i}x^{n-i}f_{m-n+i}(x)}{\sum_{i=0}^{n}a_{i}x^{n-i}}$$ (7)

Of which:

$$\tilde{p_{m}}(x)=\det[\begin{array}{lll} \left(c_{m-n+1},c_{m-n+1}\right) & (c_{m-n+1},c_{m-n+2})\ldots & \left(c_{m-n+1},c_{m+1}\right)\\ \left(c_{m-n+1},c_{m-n+2}\right) & (c_{m-n+1},c_{m-n+3})\ldots & \left(c_{m-n+1},c_{m+2}\right)\\ \vdots \\ \left(c_{m-n+1},c_{m}\right) & (c_{m-n+1},c_{m+1})\ldots .. & \left(c_{m-n+1},c_{m+n}\right)\\ \;\sum_{i=n}^{m}c_{i-n}x^{i} & \sum_{i=n-1}^{m}c_{i-n+1}x^{i} & \sum_{i=0}^{m}c_{i}x^{i} \end{array}]$$ (8)

$$\tilde{q_{n}}(x)=\det[\begin{array}{lll} \left(c_{m-n+1},c_{m-n+1}\right) & (c_{m-n+1},c_{m-n+2})\ldots & \left(c_{m-n+1},c_{m+1}\right)\\ \left(c_{m-n+1},c_{m-n+2}\right) & (c_{m-n+1},c_{m-n+3})\ldots & \left(c_{m-n+1},c_{m+2}\right)\\ \vdots \\ \left(c_{m-n+1},c_{m}\right) & (c_{m-n+1},c_{m+1})\ldots .. & \left(c_{m-n+1},c_{m+n}\right)\\ \;x^{n} & x^{n-1} & 1 \end{array}]$$ (9)

When m = n = 3, in this paper, according to the definition of padé approximation Formula (7), a new rational smooth function padé33 can be calculated by padé approximation orthogonal polynomial and determinant formula algorithms (8) and (9) to approximate the positive sign function:

$$Pade^{\prime }33(x,k)=\{\begin{array}{l} x,\;x\geq 1/k\\ \frac{7(1-k^{2}x^{2}{)}^{3}-56(1-k^{2}x^{2}{)}^{2}+112(1-k^{2}x^{2})-64}{2k({\left(1-k^{2}x^{2}\right)}^{3}-24{\left(1-k^{2}x^{2}\right)}^{2}+80(1-k^{2}x^{2})-64)}+\frac{x}{2},-1/k<x<1/k\\ 0,\;x\leq -1/k \end{array}$$ (10)

Compared with the accuracy of other smooth functions approaching the positive function, the effect of approaching the padé33 rational smooth function constructed in this paper to the positive function is shown in S2 Fig, where *k* = 10.

According to S2 Fig, sigmoid integral function, polynomials and spline functions of different degrees, padé22 function, and padé33 function constructed in this paper can approximate and smooth the positive sign function, but compared with other smooth functions, padé33 rational smooth function constructed in this paper has a significantly better approximation to the positive sign function.

### 3.2 Approximation accuracy and model convergence analysis of padé33 rational smooth function

**Theorem 1** Let *x*∈*R*, *Pad*é(*x*,*k*) be a function defined by Eq (10), then:

(1) *Pad*é33(*x*,*k*) has second-order smoothness and at the point $x=\pm \frac{1}{k},x=0$ satisfies the following conditions:

$$\left\{ \begin{array}{ll} Padé33(\frac{1}{k},k)=\frac{1}{k}, & Padé33(-\frac{1}{k},k)=0;\\ \nabla Padé33(\frac{1}{k},k)=1, & \nabla Padé33(-\frac{1}{k},k)=0;\\ \nabla^{2}Padé33(\frac{1}{k},k)=0, & \nabla^{2}Padé33(-\frac{1}{k},k)=0; \end{array}\right.$$

(2) *Pad*é33(*x*,*k*)≥*x*_+_;
(3) $Padé33{\left(x,k\right)}^{2}-x_{+}{}^{2}\leq \frac{0.0051}{k^{2}}$

**Proof:** (1) (2) easy to prove, which can also be seen from the image.

For (3), discuss according to the situation.

When $x\geq \frac{1}{k}$, or $x\leq -\frac{1}{k}$, $Padé33{\left(x,k\right)}^{2}-x_{+}{}^{2}=0\leq \frac{0.0051}{k^{2}}$ is true obviously.

When $-\frac{1}{k}\leq x\leq \frac{1}{k}$, let *t* = *kx*, then *t*∈[−1,1], so

$$\begin{array}{l} Padé33{\left(x,k\right)}^{2}-x_{+}{}^{2}={\left(\frac{7(1-k^{2}x^{2}{)}^{3}-56(1-k^{2}x^{2}{)}^{2}+112(1-k^{2}x^{2})-64}{2k({\left(1-k^{2}x^{2}\right)}^{3}-24{\left(1-k^{2}x^{2}\right)}^{2}+80(1-k^{2}x^{2})-64)}+\frac{x}{2}\right)}^{2}-x^{2}\\ \;={\left(\frac{7{\left(1-t^{2}\right)}^{3}-56{\left(1-t^{2}\right)}^{2}+112(1-t^{2})-64}{2k({\left(1-t^{2}\right)}^{3}-24{\left(1-t^{2}\right)}^{2}+80(1-t^{2})-64)}+\frac{t}{2k}\right)}^{2}-{\left(\frac{t}{k}\right)}^{2}\\ \;=\frac{1}{k^{2}}\left\{{\left(\frac{7{\left(1-t^{2}\right)}^{3}-56{\left(1-t^{2}\right)}^{2}+112(1-t^{2})-64}{2({\left(1-t^{2}\right)}^{3}-24{\left(1-t^{2}\right)}^{2}+80(1-t^{2})-64)}+\frac{t}{2}\right)}^{2}-t^{2}\right\} \end{array}$$

Let $g(t)={\left(\frac{7{\left(1-t^{2}\right)}^{3}-56{\left(1-t^{2}\right)}^{2}+112(1-t^{2})-64}{2({\left(1-t^{2}\right)}^{3}-24{\left(1-t^{2}\right)}^{2}+80(1-t^{2})-64)}+\frac{t}{2}\right)}^{2}-t^{2}$, where *t*∈[−1,1], use MATLAB software to solve the maximum value of *g*(*t*) at *t*∈[−1,1], and get max *g*(*t*) = 0.0051, thus $Padé33{\left(x,k\right)}^{2}-x_{+}{}^{2}=\frac{1}{k^{2}}g(t)\leq \frac{0.0051}{k^{2}}$.

To sum up, no matter what value *x* takes, there is always $Padé33{\left(x,k\right)}^{2}-x_{+}{}^{2}\leq \frac{0.0051}{k^{2}}$.

**Theorem 2** let ***A***∈***R***^*m*×*n*^ and *b*∈***R***^*n*×1^.The real function *h*(***x***):*R*^*n*^→*R* and *g*(***x***,*k*):*R*^*n*^×*N*→*R* are defined as follows:

$$h(x)=\frac{1}{2}{\left\|{\left(Ax-b\right)}_{+}\right\|}_{2}^{2}+\frac{1}{2}{\left\|x\right\|}_{2}^{2}$$ (11)

$$g(x,k)=\frac{1}{2}{\left\|Padé33{\left(Ax-b,k\right\|}_{+}\right)}_{2}^{2}+\frac{1}{2}{\left\|x\right\|}_{2}^{2}$$ (12)

Where *k*>0, *Pad*é33(*x*,*k*) is defined by Formula (10), then the following conclusion is true:

1. *h*(***x***) and *g*(***x***,*k*) are strongly convex functions;
2. The optimization problems $\underset{x}{\min}h(x)$ and $\underset{x}{\min}g(x,k)$ have unique solutions, which are recorded as $\bar{x}$ and ${\bar{x}}^{k}$ respectively;
3. For any *k*≥1, ${\left\|{\bar{x}}^{k}-\bar{x}\right\|}_{2}^{2}\leq \frac{0.0026m}{k^{2}}$ holds, where *m* is the number of training samples;
4. $\underset{k\to \infty }{\lim}{\bar{x}}^{k}=\bar{x}$。

**Proof:** the proof idea of this theorem is similar to the method involved in literature [7]

1. Because of the inherent strong convexity of ${\left\|•\right\|}_{2}^{2}$, according to the formulas of *h*(***x***) and *g*(***x***,*k*), it can be concluded that these two functions also conform to strong convexity, so they are strong convex functions.
2. According to the formulas of *h*(***x***) and *g*(***x***,*k*), the corresponding level sets can be set separately as follows: $L_{\nu }(h(x))=\{x|x\in R^{n}),h(x)\leq \nu \}.$ $L_{\nu }(g(x,k))=\{x|x\in R^{n}),g(x,k)\leq \nu \}$. Because of $Padé33(x,k)\geq x_{+}$, for any *v*≥0, they satisfy $L_{\nu }(g(x,k))\subset L_{\nu }(h(x))\subset \{x{\left\|x\right\|}_{2}^{2}\leq 2\nu \}$. Therefore, both min *h*(***x***) and min *g*(***x***,*k*) are compact sets in ***R***^***n***^ space, so the optimization problems min *h*(***x***) and min *g*(***x***,*k*) have optimal solutions. In addition, for any *k*∈***Z***^+^, *h*(***x***) and *g*(***x***,*k*) also satisfy strong convexity, so $\underset{x\in R^{n}}{\min}h(x)$ and $\underset{x\in R^{n}}{\min}g(x,k)$ have unique solutions, which can be recorded as $\bar{x}$ and ${\bar{x}}^{k}$。
3. Let the unique solutions of min *h*(***x***) a and min *g*(***x***,*k*) be $\bar{x}$ and ${\bar{x}}^{k}$ respectively, which is obvious: $h({\bar{x}}^{k})-h(\bar{x})\geq \nabla h(\bar{x})({\bar{x}}^{k}-\bar{x})+\frac{1}{2}{\left\|{\bar{x}}^{k}-\bar{x}\right\|}_{2}^{2}=\frac{1}{2}{\left\|{\bar{x}}^{k}-\bar{x}\right\|}_{2}^{2}$ $g(\bar{x},k)-g({\bar{x}}^{k},k)\geq \nabla g({\bar{x}}^{k},k)(\bar{x}-{\bar{x}}^{k})+\frac{1}{2}{\left\|{\bar{x}}^{k}-\bar{x}\right\|}_{2}^{2}=\frac{1}{2}{\left\|{\bar{x}}^{k}-\bar{x}\right\|}_{2}^{2}$,

Add the above two equations to get:

$$\begin{array}{l} {\left\|{\bar{x}}^{k}-\bar{x}\right\|}_{2}^{2}\leq (h({\bar{x}}^{k})-h(\bar{x}))+(g(\bar{x},k)-g({\bar{x}}^{k},k))\\ \;\leq (g(\bar{x},k)-h(\bar{x}))-(g({\bar{x}}^{k},k)-h({\bar{x}}^{k}))\\ \;\leq g(\bar{x},k)-h(\bar{x})\\ \;\leq \frac{1}{2}{\left\|Padé33{\left(Ax-b,k\right)}_{+}\right\|}_{2}^{2}-\frac{1}{2}{\left\|{\left(Ax-b\right)}_{+}\right\|}_{2}^{2} \end{array}$$

From (3) $Padé33{\left(x,k\right)}^{2}-x_{+}{}^{2}\leq \frac{0.0051}{k^{2}}$ of Theorem 1:

$${\left\|{\bar{x}}^{k}-\bar{x}\right\|}_{2}^{2}\leq \frac{1}{2}{\left\|Padé33{\left(Ax-b,k\right\|}_{+}\right)}_{2}^{2}-\frac{1}{2}{\left\|{\left(Ax-b\right)}_{+}\right\|}_{2}^{2}\leq \frac{0.0051m}{2k^{2}}=\frac{0.0026m}{k^{2}}$$

(4) From (3): ${\left\|{\bar{x}}^{k}-\bar{x}\right\|}_{2}^{2}\leq \frac{0.0026m}{k^{2}}$, so $\underset{k\to \infty }{\lim}{\left\|{\bar{x}}^{k}-\bar{x}\right\|}_{2}^{2}\leq \underset{k\to \infty }{\lim}\frac{0.0026m}{k^{2}}=0$

Therefore $\underset{k\to \infty }{\lim}{\bar{x}}^{k}=\bar{x}$.

From theorem 2, it can be found that when the smoothing coefficient *k* tends to infinity, the unique solution based on padé33 SSVM can be approximate to the solution of the original optimization model (4). Therefore, the solution of padé33 SSVM established in this paper meets the convergence. In addition, according to Table 1 and S2 Fig, it can be found that the Padé33 rational smooth function constructed in this paper is closer to the positive sign function than the previous smooth function, and its approximation accuracy is one order of magnitude higher than the original Sigmoid integral function. At the same time, in terms of convergence speed, the Padé33-SSVM established in this paper is also faster than the existing SSVM, and two orders of magnitude higher than the Sigmoid-SSVM.

**Table 1:** Approximation accuracy of different smooth functions and the convergence rate of the model.

Smooth support vector machine(SSVM)	Smooth function *f*_smooth_(*x*,*k*)	Approximation accuracy $f_{smooth}{\left(x,k\right)}^{2}-x_{+}^{2}\leq$	Convergence speed $\|{\bar{x}}^{k}-\bar{x}{\|}_{2}^{2}\leq$
Sigmoid-SSVM	$x+\frac{1}{k}\log(1+e^{-kx})$	0.6927/*k*^2^.	0.3463*m*/*k*^2^
P2-SSVM	$\frac{k}{4}x^{2}+\frac{x}{2}+\frac{1}{4k}$.	0.0909/*k*^2^	0.0455*m*/*k*^2^
P4-SSVM	$-\frac{1}{16k}{\left(kx+1\right)}^{3}(kx-3)$	0.0526/*k*^2^	0.0263*m*/*k*^2^
T3-SSVM	$\{\begin{array}{l} \frac{k^{2}}{6}x^{3}+\frac{k}{2}x^{2}+\frac{1}{2}x+\frac{1}{6k},-\frac{1}{k}<x<0\\ -\frac{k^{2}}{6}x^{3}+\frac{k}{2}x^{2}+\frac{1}{2}x+\frac{1}{6k},0\leq x<\frac{1}{k} \end{array}$	0.04167/*k*^2^	0.0208*m*/*k*^2^
T5-SSVM	$\{\begin{array}{l} -\frac{k^{4}}{10}x^{5}-\frac{k^{3}}{4}x^{4}+\frac{k}{2}x^{2}+\frac{1}{2}x+\frac{3}{20k},-\frac{1}{k}<x<0\\ \frac{k^{4}}{10}x^{5}-\frac{k^{3}}{4}x^{4}+\frac{k}{2}x^{2}+\frac{1}{2}x+\frac{3}{20k},0\leq x<\frac{1}{k} \end{array}$	0.03333/*k*^*2*^	0.0167*m*/*k*^2^
Padé22-SSVM	$\frac{1}{2k}\cdot \frac{1+10k^{2}x^{2}+5k^{4}x^{4}}{5+10k^{2}x^{2}+k^{4}x^{4}}+\frac{x}{2}$	0.0139/*k*^2^	0.0069*m*/*k*^2^
Padé33-SSVM	$\frac{7(1-k^{2}x^{2}{)}^{3}-56(1-k^{2}x^{2}{)}^{2}+112(1-k^{2}x^{2})-64}{2k({\left(1-k^{2}x^{2}\right)}^{3}-24{\left(1-k^{2}x^{2}\right)}^{2}+80(1-k^{2}x^{2})-64)}+\frac{x}{2}$	0.0051/*k*^2^	0.0026*m*/*k*^2^

## 4. A training algorithm for smooth support vector machines

In model (5), the nonlinear classification problem can be solved only by introducing a kernel function. Therefore, model (5) still maintains strong convexity and smoothness for any kernel function, and its convergence still holds in this nonlinear case. Therefore, for the smoothed model (5), the BFGS Armijo optimization algorithm [25] can be directly used to classify its solution. To make better use of this algorithm, this paper unifies the notation, denoting the objective function of the model (5) as *φ*(*w*,*b*), its first-order derivation as ∇*φ*(*w*,*b*), the solution accuracy of the algorithm itself is mainly *ε* = 10^−5^, and the number of iterative steps is denoted as *i*. Thus, the BFGS-Armijo optimization algorithm is used to give the calculation steps when the objective function is first-order smooth, as follows:

**Step 1:** Initialization $(\omega_{0},b_{0})=u_{0}\in R^{n+1},H_{0}=I,$ set max = 1000, *i* = 0,and *ε* = 10^−5^.

**Step 2:** If *i*≤max, calculate *v*_*i*_ = ∇*φ*(*u*_*i*_).

**Step 3:**If ${\left\|v_{i}\right\|}_{2}^{2}\leq \varepsilon$, then stop, the optimal solution of model (5) can be obtained as *u*_*i*_ = (*ω*_*i*_,*b*_*i*_). Otherwise, calculate *d*_*i*_ = −*H*_*i*_*v*_*i*_, then go to step 4;

**Step 4:**Performe a one-dimensional search along the direction *d*_*i*_ to get the step factor *α*_*i*_>0:

Let

$$\begin{array}{l} u_{i+1}=u_{i}+\alpha_{i}d_{i},\\ s_{i}=u_{i+1}-u_{i}=-\alpha_{i}H_{i}v_{i}. \end{array}$$

Then calculate

$$\begin{array}{l} \varphi_{i+1}=\varphi (u_{i+1}),\\ \nabla \varphi_{i+1}=\nabla \varphi (u_{i+1}),\\ y^{i}=\nabla \varphi_{i+1}-\nabla \varphi_{i}. \end{array}$$

**Step 5:** Correct *H*_*i*_ to get *H*_*i*+1_;

$$H_{i+1}=(I-\frac{s_{i}{\left(y_{i}\right)}^{T}}{{\left(s_{i}\right)}^{T}y_{i}})H_{i}(I-\frac{y_{i}{\left(s_{i}\right)}^{T}}{{\left(s_{i}\right)}^{T}y_{i}})+\frac{s_{i}{\left(s_{i}\right)}^{T}}{{\left(s_{i}\right)}^{T}y_{i}},$$

**Step 6:** Then let *i* = *i*+1, go to step 2; where *φ*(*u*_*i*_) is defined according to model (4), and *α*_*i*_ can be obtained according to the following formula:

$$\left\{ \begin{array}{l} \varphi (u_{i}+\alpha_{i}d_{i})\leq \varphi (u_{i})+\rho {\left(v_{i}\right)}^{T}u_{i},\\ \varphi (u_{i}+\alpha_{i}d_{i})\geq \varphi (u_{i})+(1-\rho ){\left(v_{i}\right)}^{T}u_{i},\\ u_{i}=\alpha_{i}d_{i},0<\rho <\frac{1}{2}. \end{array}\right.$$

## 5. Numerical experiments

To verify the application effect of padé33-SSVM model constructed in this paper in practical classification problems, based on the training and testing data sets of the UCI machine learning database, the training and prediction experiments of the classifier were designed for the heart disease data set. The experiment in Section 5.1 uses different SSVM models to train on the heart disease data set with different scales, and compares them with classical SVM and LSSVM. The main purpose of the experiment in Section 5.2 is to verify whether the Padé33-SSVM can obtain more accurate classification accuracy in prediction under different numbers of training samples. In addition, simulation experiment 5.3 is mainly aimed at the classification machine training experiment of different SSVMS with kernel function under different datasets of different sizes, and mainly verifies the applicability of the proposed model in classification problems under large datasets.

### 5.1 Classifier training based on heart disease data

**Experiment 1:** The heart disease dataset was obtained from the Cleverland Clinic Foundation, with sample data available from the UCI Machine Learning Database. There are a total of 270 sample data, and each sample includes 13 attributes such as gender, age, diastolic blood pressure, cholestoral content per deciliter of plasma, chest pain category, and blood sugar. All the sample data on heart disease status are divided into two categories: presence and absence.

Due to the large individual differences of each patient and the different speed of heart rate, the value range is significantly different. At the same time, in view of the redundancy and noise of the real data provided by the hospital, it is necessary to normalize the data to make the data value between [–1,1]. By normalizing the data, the redundancy of sample data can be eliminated, and the low computational efficiency under large sample data can also be overcome.

To compare the classification effects of different smooth support vector machine models, the generalization ability of the classifier is generally used as an index. The generalization ability of the classifier is usually measured by the accuracy of the training samples, and the time CPU consumed by the algorithm is recorded as the training time (s). The experiment in this section adopts model (5), in which the function *f*(*x*) adopts Sigmoid integral function, quadratic and quartic polynomial, cubic and quintic spline functions, Padé22 and Padé33 rational smooth function constructed in this paper. By solving the smooth support vector machine (padé33-SSVM) based on padé33 rational smooth function, the decision function is *g*(*x*) = sgn(*ω*^*T*^*x*−*b*), where $\omega =[0.01227\;‐0.36243\;‐0.19132\;‐0.00526\;‐0.00168\;0.18655\;‐0.10120\;0.01017\;‐0.25288\;‐0.12861\;‐0.05915\;‐0.37907\;‐0.12470{]}^{T},\;b=\;-1.0781$. Different SSVM models are used to train the data sets with inconsistent scales on the heart disease data sets, and BFGS Armijo algorithm is used to solve Eq (5). The performance indicators obtained from the solution are shown in Table 2.

**Table 2:** Comparison of different SSVMS with SVM and LSSVM at different scales.

performance SSVM	Classification accuracy (percentage) M = 130	CPU Training time (s)	Classification accuracy (percentage) M = 270	CPU Training time (s)
SVM	83.850	6.817	85.560	10.043
LSSVM	81.539	0.032	84.815	0.055
Sigmoid-SSVM	83.077	0.089	84.074	0.943
P2-SSVM	84.184	0.073	84.444	0.686
P4-SSVM	84.444	0.032	84.815	0.751
T3-SSVM	84.615	0.089	85.185	0.689
T5-SSVM	84.615	0.069	85.185	0.670
Padé22-SSVM	84.615	0.074	85.556	0.517
**Padé33-SSVM**	**85.556**	**0.059**	**86.296**	**0.462**

Two conclusions can be drawn from the experimental results in Table 2. On the one hand, under different training scales, compared with classical SVM, the Padé33-SSVM constructed in this paper has an absolute advantage in training accuracy and time. Compared with LSSVM, the training accuracy in this paper is also relatively high. On the other hand, when different SSVM models are used for classification training under different data scales, the larger the scale, the higher the accuracy and the longer the time-consuming. In addition, under the same data scale, the smooth model established when the padé33 rational smooth function constructed in this paper approximates the positive function can obtain higher classification accuracy in the classification of heart disease data. Therefore, the smooth support vector machine (padé33-SSVM) based on the padé33 rational smooth function constructed in this paper can obtain higher accuracy when it is used for classification training of heart disease data. The numerical experiment shows that we can get the decision function through Padé33-SSVM, and can obtain more accurate judgment when we predict the data of heart diseases with 13 attributes.

### 5.2 Classifier prediction based on heart disease data

**Experiment 2:** According to 5.1, the total sample data about heart disease is 270, and each sample data has 13 attributes. This experiment mainly verifies whether the padé33-SSVM established in this paper can obtain more accurate classification accuracy in prediction. The experiment divides the sample data into two steps. The first step is to obtain the classification accuracy, training time, and decision function by randomly searching the data for training; The second step is to predict the remaining test data through the decision function of one stage, and the accuracy and time of prediction can be obtained. The experiment adopts model (5), in which the smooth function *f*(*x*) is the padé33 rational smooth function constructed in this paper. The classification effect is shown in Table 3 by solving padé33-SSVM through the optimization algorithm:

**Table 3:** Classification and test comparison of padé33-SSVM under different training sample functions.

samples	*C* = 2 Train Predict		*C* = 4 Train Predict		*C* = 10 Train Predict		*C* = 16 Train Predict		*C* = 20 Train Predict	
10	100 0.088	65.385 0.025	100 0.112	65 0.028	100 0.352	65.769 0.028	100 0. 319	66.154 0.028	100 0.228	65.769 0.026
30	96.667 0.104	69.167 0.027	100 0.153	67.5 0.026	100 0.285	69.583 0.030	100 0.474	69.583 0.029	100 0.472	69.583 0.036
50	96 0.133	75.909 0.016	96 0.142	74.546 0.025	96 0.197	74.546 0.023	98 0.223	75 0.033	98 0.376	74.091 0.023
70	88.571 0.127	79.5 0.028	88.571 0.105	79 0.016	92.857 0.166	77 0.027	91.429 0.218	76.5 0.017	91.429 0.246	77.5 0.026
90	88.889 0.127	81.111 0.014	90 0.162	80.556 0.021	90 0.227	78.889 0.033	90 0.179	78.333 0.027	90 0.266	78.889 0.033
130	85.385 0.203	82.143 0.020	85.385 0.219	82.143 0.034	86.154 0.297	82.143 0.032	86.154 0.237	81.429 0.026	86.154 0.417	81.429 0.023
150	86 0.251	82.5 0.021	86.667 0.291	82.5 0.013	86.667 0.316	82.5 0.017	85.333 0.355	81.667 0.013	85.333 0.418	81.833 0.017
210	88.095 0.370	83.333 0.013	87.619 0.448	83.333 0.009	88.095 0.483	81.667 0.014	88.095 0.471	81.667 0.017	87.619 0.528	81.667 0.022
240	86.667 0.490	86.667 0.010	86.667 0.686	86.667 0.017	86.25 0.678	86.667 0.031	86.25 0.869	86.667 0.014	86.25 1.046	86.667 0.017

The experimental results are shown in Table 3. With the increase of the number of training samples, the more accurate the decision function is, and then when predicting the remaining sample data, the higher the accuracy is. This is also consistent with the reality, that is, the larger the sample data size, the more accurate the decision function can be trained. In addition, the decision function trained is also different due to the different number of training samples, and after the padé33-SSVM training constructed in this paper, the model can adjust the parameter C value to obtain the function with high training classification accuracy for the second stage of prediction experiment. Therefore, for the training samples of heart disease under the scale of large data, a more accurate decision function can be obtained after the Padé33-SSVM classification training in this paper. As a result, the data of heart disease can be diagnosed and predicted, the potential patients with heart disease can be predicted and treated early, and the early warning can be given to those who do not suffer from heart disease.

### 5.3 Classifier training based on large data sets

**Experiment 3:** The experiment in this section solves the classification problem of large data sets by solving model (5), and the kernel function selected is Gaussian kernel function [14] $K(x,y)=\exp(\frac{-{\left\|x-y\right\|}^{2}}{2\sigma^{2}})$. The sample datasets used in this experiment include Banknote authentication, EEG Eye Status, QSAR biodegradation, Crowdsourced mapping and Diabetic Retinopathy Debrecen datasets, which are all from the UCI database (http://archive.ics.uci.edu/ml/datasets.php?format=&task=cla&att=&area=&numAtt=&numIns=&type=&sort=nameUp&view=table). The banknote authentication data is 1372 * 4, extracted from the image used to evaluate the banknote authentication program, and all data samples are divided into two categories. The original EEG state data is 14,980 * 20 dimensions, and the data are divided into two categories: ’1’ for closed eyes and ’0’ for open eyes; QSAR biodegradation data is 1055 * 41, and 1055 chemical samples are divided into 2 categories; The original mapping data of crowdsourcing is 10546 * 28, and the crowdsourcing data is divided into six categories. To facilitate calculation, two categories (farm and forest) data are randomly searched as training data. The Debrecen data of diabetes retinopathy is 1151 * 20. The data set contains features extracted from the Messidor image set to predict whether the image contains signs of diabetes retinopathy, and these 1151 chemicals are divided into two categories. In the experiment, 1372, 1500, 1055, 2000 and 1151 samples were randomly selected from the above dataset for classification training. The results are shown in Table 4. The data in each column is the training accuracy rate (%), and the training time (s) is in the parentheses in the second row.

**Table 4:** Comparison of different SSVMS with SVM and LSSVM in different data sets.

Data sets Performance SSVM	Banknote = 1372*4	EEG = 1500*20	QSAR = 1055*41	Crowdsourced = 2000*28	Diabetic = 1151*20
SVM	90.690 (336.294)	62.330 (485.430)	85.010 (1863.9)	92.900 (5655.1)	69.750 (3531.0)
LSSVM	87.670 (353.127)	61.470 (0.912)	82.200 (3.134)	92.100 (2.108)	63.330 (1.348)
Sigmoid-SSVM	88.484 (353.127)	61.000 (283.030)	83.212 (279.697)	89.850 (853.942)	57.863 (254.657)
P2-SSVM	88.484 (299.069)	61.733 (243.039)	83.318 (280.922)	91.150 (879.012)	60.016 (256.051)
P4-SSVM	88.776 (297.164)	62.000 (273.971)	83.697 (285.665)	91.350 (927.105)	60.035 (255.993)
T3-SSVM	88.921 (294.272)	62.133 (244.845)	83.697 (342.235)	91.250 (899.858)	64.987 (256.087)
T5-SSVM	88.994 (298.486)	62.133 (245.261)	83.886 (311.801)	91.350 (939.453)	64.992 (256.884)
Padé22-SSVM	89.723 (295.753)	62.333 (250.239)	84.171 (396.057)	92.200 (852.956)	65.682 (256.969)
**Padé33-SSVM**	**89.923** **(276.532)**	**62.415** **(228.632)**	**84.435** **(280.362)**	**92.200** **(836.634)**	**68.215** **(250.351)**

According to Table 4, under different data set sizes, compared with classical SVM and LSSVM, Pade33-SSVM constructed in this paper has a slightly lower training accuracy than SVM, but it takes a shorter time and speeds up the calculation speed. Compared with LSSVM, although the training time is relatively long, the training accuracy is significantly higher than that of LSSVM. Therefore, compared with SSVM and LSSVM, the Padé33-SSVM constructed in this paper is not only suitable for the training and prediction of heart disease data, but also more suitable for the classification of data sets of different sizes. In general, when different SSVMs with kernel function are used for classification training, the larger the scale is, the longer the time is. Under the same data scale, higher classification accuracy can be obtained when using Padé33 rational smooth function to approximate the positive sign function. Therefore, Padé33-SSVM is not only applicable to the classification of small-scale data, but also for the case of large data sets.

## 6. Conclusions

To overcome the non-differentiability of SVM, first, based on the Padé type approximation orthogonal polynomial definition and determinant algorithm, this paper constructs a Padé33 rational smooth function to approximate the positive sign function. Secondly, Theorem 1 and Theorem 2 are proposed to prove that the smoothness accuracy of the Padé33 function is significantly higher than that of the existing smooth functions, and the convergence performance of padé33-SSVM is better than that of other SSVMs. Finally, the new smooth model established in this paper is used to diagnose heart disease. The experimental results show that padé33-SSVM has better classification ability and higher classification accuracy than the other four SSVMs. Therefore, the padé33-SSVM constructed in this paper is of great significance in real life, not limited to the prediction of heart disease.

In the future, the padé33-SSVM model established in this paper can be used to predict the actual classification problems such as broader medical diagnosis, customer churn in company management, and whether the enterprise’s finance is in trouble.

## Supporting information

S1 Fig Schematic diagram of linear separable support vector machine. (TIF)

S2 Fig Effect diagram of different smooth functions approaching positive sign function (k = 10). (TIF)

S1 Table Approximation accuracy of different smooth functions and the convergence rate of the model. (PDF)

S2 Table Comparison of different SSVMS with SVM and LSSVM at different scales. (PDF)

S3 Table Classification and test comparison of padé33-SSVM under different training sample functions. (PDF)

S4 Table Comparison of different SSVMS with SVM and LSSVM in different data sets. (PDF)
