# Supplementary figures and images for: Construction of a new smooth support vector machine model and its application in heart disease diagnosis
Source: PLoS One. 2023 Feb 9;18(2):e0280804. doi: 10.1371/journal.pone.0280804 (PMC9910651; doi:10.1371/journal.pone.0280804)

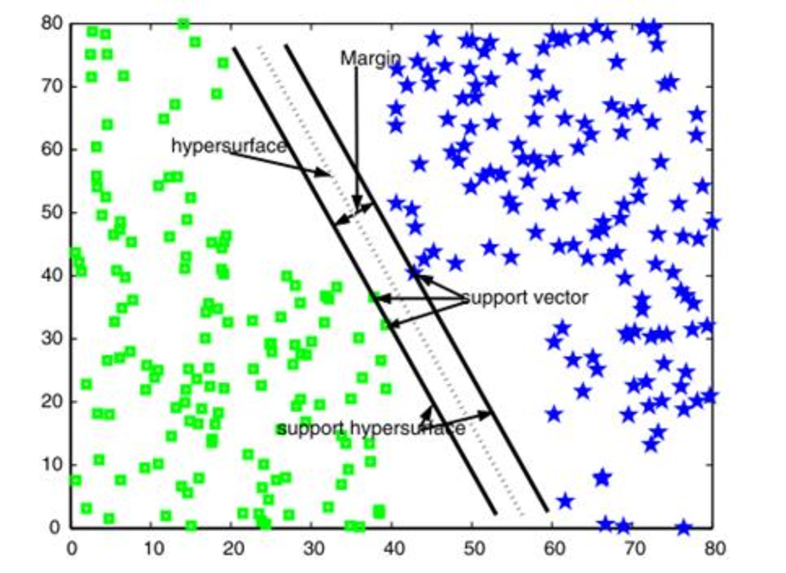

Supplement: S1 Fig — (TIF) [file pone.0280804.s001.tif]

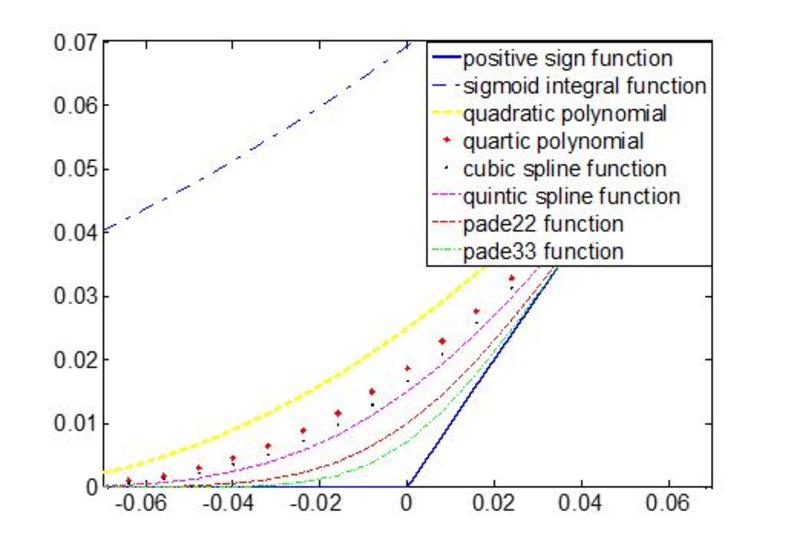

Supplement: S2 Fig — (TIF) [file pone.0280804.s002.tif]
