# Supplementary material for: Construction of a new smooth support vector machine model and its application in heart disease diagnosis
Source: PLoS One. 2023 Feb 9;18(2):e0280804. doi: 10.1371/journal.pone.0280804 (PMC9910651; doi:10.1371/journal.pone.0280804)
Supplement: S1 Table — (PDF) [file pone.0280804.s003.pdf]

S1 Table. Approximation accuracy of different smooth functions and the convergence rate of the model.

Smooth support vector machine （SSVM）	Smooth function $f_{smooth}\left(x,k\right)$	Approximation accuracy $f_{smooth}\left(x,k\right)^2 - x_+^2 \le$	Convergence speed $\left\Vert \bar{x}^k - \bar{x} \right\Vert_2^2 \le$
Sigmoid-SSVM	$x+\frac{1}{k}\log\left(1+e^{-kx}\right)$	$0.6927/k^2$	$0.3463m/k^2$
P2-SSVM	$\frac{k}{4}x^2+\frac{x}{2}+\frac{1}{4k}$	$0.0909/k^2$	$0.0455m/k^2$
P4-SSVM	$-\frac{1}{16k}\left(kx+1\right)^3\left(kx-3\right)$	$0.0526/k^2$	$0.0263m/k^2$
T3-SSVM	$\begin{cases}\frac{k^2}{6}x^3+\frac{k}{2}x^2+\frac{1}{2}x+\frac{1}{6k}, -\frac{1}{k}<x<0\\ -\frac{k^2}{6}x^3+\frac{k}{2}x^2+\frac{1}{2}x+\frac{1}{6k}, 0\le x<\frac{1}{k}\end{cases}$	$0.04167/k^2$	$0.0208m/k^2$
T5-SSVM	$\begin{cases}-\frac{k^4}{10}x^5-\frac{k^3}{4}x^4+\frac{k}{2}x^2+\frac{1}{2}x+\frac{3}{20k}, -\frac{1}{k}<x<0\\ \frac{k^4}{10}x^5-\frac{k^3}{4}x^4+\frac{k}{2}x^2+\frac{1}{2}x+\frac{3}{20k}, 0\le x<\frac{1}{k}\end{cases}$	$0.03333/k^2$	$0.0167m/k^2$
Padé22-SSVM	$\frac{1}{2k}\cdot\frac{1+10\mathrm{k}^2x^2+5k^4x^4}{5+10k^2x^2+k^4x^4}+\frac{x}{2}$	$0.0139/k^2$	$0.0069m/k^2$
Padé33-SSVM	$\frac{7(1\text{-}k^2x^2)^3-56(1\text{-}k^2x^2)^2+112(1\text{-}k^2x^2)-64}{2k\left((1\text{-}k^2x^2)^3-24(1\text{-}k^2x^2)^2+80(1\text{-}k^2x^2)\text{-}64\right)}+\frac{x}{2}$	$0.0051/k^2$	$0.0026m/k^2$
