# Supplementary material for: Construction of a new smooth support vector machine model and its application in heart disease diagnosis
Source: PLoS One. 2023 Feb 9;18(2):e0280804. doi: 10.1371/journal.pone.0280804 (PMC9910651; doi:10.1371/journal.pone.0280804)
Supplement: S2 Table — (PDF) [file pone.0280804.s004.pdf]

S2 Table. Comparison of different SSVMS with SVM and LSSVM at different scales.

performance / SSVM	Classification accuracy (percentage) M=130	CPU Training time (s)	Classification accuracy (percentage) M=270	CPU Training time (s)
SVM	83.850	6.817	85.560	10.043
LSSVM	81.539	0.032	84.815	0.055
Sigmoid-SSVM	83.077	0.089	84.074	0.943
P2-SSVM	84.184	0.073	84.444	0.686
P4-SSVM	84.444	0.032	84.815	0.751
S3-SSVM	84.615	0.089	85.185	0.689
S5-SSVM	84.615	0.069	85.185	0.670
Padé22-SSVM	84.615	0.074	85.556	0.517
**Padé33-SSVM**	**85.556**	**0.059**	**86.296**	**0.462**
