# Supplementary material for: Construction of a new smooth support vector machine model and its application in heart disease diagnosis
Source: PLoS One. 2023 Feb 9;18(2):e0280804. doi: 10.1371/journal.pone.0280804 (PMC9910651; doi:10.1371/journal.pone.0280804)
Supplement: S3 Table — (PDF) [file pone.0280804.s005.pdf]

**S3 Table. Classification and test comparison of padé33-SSVM under different training sample functions.**

| samples | $C=2$ | | $C=4$ | | $C=10$ | | $C=16$ | | $C=20$ | |
|---|---|---|---|---|---|---|---|---|---|---|
| | Train | Predict | Train | Predict | Train | Predict | Train | Predict | Train | Predict |
| 10 | 100 | 65.385 | 100 | 65 | 100 | 65.769 | 100 | 66.154 | 100 | 65.769 |
| | 0.088 | 0.025 | 0.112 | 0.028 | 0.352 | 0.028 | 0. 319 | 0.028 | 0.228 | 0.026 |
| 30 | 96.667 | 69.167 | 100 | 67.5 | 100 | 69.583 | 100 | 69.583 | 100 | 69.583 |
| | 0.104 | 0.027 | 0.153 | 0.026 | 0.285 | 0.030 | 0.474 | 0.029 | 0.472 | 0.036 |
| 50 | 96 | 75.909 | 96 | 74.546 | 96 | 74.546 | 98 | 75 | 98 | 74.091 |
| | 0.133 | 0.016 | 0.142 | 0.025 | 0.197 | 0.023 | 0.223 | 0.033 | 0.376 | 0.023 |
| 70 | 88.571 | 79.5 | 88.571 | 79 | 92.857 | 77 | 91.429 | 76.5 | 91.429 | 77.5 |
| | 0.127 | 0.028 | 0.105 | 0.016 | 0.166 | 0.027 | 0.218 | 0.017 | 0.246 | 0.026 |
| 90 | 88.889 | 81.111 | 90 | 80.556 | 90 | 78.889 | 90 | 78.333 | 90 | 78.889 |
| | 0.127 | 0.014 | 0.162 | 0.021 | 0.227 | 0.033 | 0.179 | 0.027 | 0.266 | 0.033 |
| 130 | 85.385 | 82.143 | 85.385 | 82.143 | 86.154 | 82.143 | 86.154 | 81.429 | 86.154 | 81.429 |
| | 0.203 | 0.020 | 0.219 | 0.034 | 0.297 | 0.032 | 0.237 | 0.026 | 0.417 | 0.023 |
| 150 | 86 | 82.5 | 86.667 | 82.5 | 86.667 | 82.5 | 85.333 | 81.667 | 85.333 | 81.833 |
| | 0.251 | 0.021 | 0.291 | 0.013 | 0.316 | 0.017 | 0.355 | 0.013 | 0.418 | 0.017 |
| 210 | 88.095 | 83.333 | 87.619 | 83.333 | 88.095 | 81.667 | 88.095 | 81.667 | 87.619 | 81.667 |
| | 0.370 | 0.013 | 0.448 | 0.009 | 0.483 | 0.014 | 0.471 | 0.017 | 0.528 | 0.022 |
| 240 | 86.667 | 86.667 | 86.667 | 86.667 | 86.25 | 86.667 | 86.25 | 86.667 | 86.25 | 86.667 |
| | 0.490 | 0.010 | 0.686 | 0.017 | 0.678 | 0.031 | 0.869 | 0.014 | 1.046 | 0.017 |
