# Supplementary material for: Construction of a new smooth support vector machine model and its application in heart disease diagnosis
Source: PLoS One. 2023 Feb 9;18(2):e0280804. doi: 10.1371/journal.pone.0280804 (PMC9910651; doi:10.1371/journal.pone.0280804)
Supplement: S4 Table — (PDF) [file pone.0280804.s006.pdf]

S4 Table. Comparison of different SSVMS with SVM and LSSVM in different data sets.

Data sets / Performance / SSVM	Banknote =1372*4	EEG =1500*20	QSAR =1055*41	Crowdsourced =2000*28	Diabetic =1151*20
SVM	90.690 (336.294)	62.330 (485.430)	85.010 (1863.9)	92.900 (5655.1)	69.750 (3531.0)
LSSVM	87.670 (353.127)	61.470 (0.912)	82.200 (3.134)	92.100 (2.108)	63.330 (1.348)
Sigmoid-SSVM	88.484 (353.127)	61.000 (283.030)	83.212 (279.697)	89.850 (853.942)	57.863 (254.657)
P2-SSVM	88.484 (299.069)	61.733 (243.039)	83.318 (280.922)	91.150 (879.012)	60.016 (256.051)
P4-SSVM	88.776 (297.164)	62.000 (273.971)	83.697 (285.665)	91.350 (927.105)	60.035 (255.993)
T3-SSVM	88.921 (294.272)	62.133 (244.845)	83.697 (342.235)	91.250 (899.858)	64.987 (256.087)
T5-SSVM	88.994 (298.486)	62.133 (245.261)	83.886 (311.801)	91.350 (939.453)	64.992 (256.884)
Padé22-SSVM	89.723 (295.753)	62.333 (250.239)	84.171 (396.057)	92.200 (852.956)	65.682 (256.969)
**Padé33-SSVM**	**89.923** (276.532)	**62.415** (228.632)	**84.435** (280.362)	**92.200** (836.634)	**68.215** (250.351)
